# Estigma en personas sin hogar y atención en salud: reflexiones desde un programa Housing First en Barcelona, España

**DOI:** 10.18294/sc.2024.4826

**Published:** 2024-06-05

**Authors:** Marta Llobet-Estany, Mercedes Serrano-Miguel, Araceli Muñoz, Adela Boixadós-Porquet, Belén Campomar

**Affiliations:** 1 Doctora en Sociología. Docente e investigadora, Unitat de Formació i Recerca - Escola de Treball Social, Universitat de Barcelona, España. mllobet@ub.edu Universitat de Barcelona Unitat de Formació i Recerca - Escola de Treball Social Universitat de Barcelona Spain mllobet@ub.edu; 2 Doctora en Antropología Social. Docente e investigadora, Unitat de Formació i Recerca - Escola de Treball Social, Universitat de Barcelona, España. mercedes.serrano@ub.edu Universitat de Barcelona Unitat de Formació i Recerca - Escola de Treball Social Universitat de Barcelona Spain mercedes.serrano@ub.edu; 3 Doctora en Antropología Social. Docente e investigadora, Unitat de Formació i Recerca -Escola de Treball Social, Universitat de Barcelona, España. aracelimunoz67@ub.edu Universitat de Barcelona Unitat de Formació i Recerca -Escola de Treball Social Universitat de Barcelona Spain aracelimunoz67@ub.edu; 4 Licenciada en Sociología, diplomada en Trabajo Social. Docente e investigadora, Unitat de Formació i Recerca - Escola de Treball Social, Universitat de Barcelona, España. aboixados@ub.edu Universitat de Barcelona Unitat de Formació i Recerca - Escola de Treball Social Universitat de Barcelona Spain aboixados@ub.edu; 5 Licenciada en Relaciones Internacionales, Especialista en Política, Evaluación y Gerencia Social. Investigadora, Universitat de Barcelona, España. belen.campomar@gmail.com Universitat de Barcelona Universitat de Barcelona Spain belen.campomar@gmail.com

**Keywords:** Personas sin Hogar, Estigma Social, Atención a la Salud, España

## Abstract

El tránsito por el sinhogarismo está asociado a procesos de fuerte estigmatización que, en muchas ocasiones, tienen su reflejo en el trato que reciben por parte de las y los profesionales y del propio sistema de atención en salud. Este artículo tiene como objetivo analizar las experiencias que tuvieron en el sistema sanitario las y los participantes de un programa para personas sin hogar en Barcelona llamado Primer la Llar, y cómo el estigma que sufren estas personas llega a condicionar los procesos de atención. Dicho programa sigue el modelo Housing First, una intervención social que propone la entrada a una vivienda sin condiciones previas a personas con largas trayectorias de vida en la calle, que sufren trastornos mentales graves y/o adicciones. A partir de entrevistas individuales con 20 participantes, realizadas entre 2016 y 2020, se observa que, en determinados casos, el ingreso en el programa, la disponibilidad de una vivienda, el soporte de profesionales y el desarrollo de estrategias propias tuvieron efectos positivos en la mejora de su salud, aunque continúan percibiendo actitudes discriminatorias en algunos espacios médicos. Se plantea la necesidad de que la transformación respecto a la estigmatización sea entendida en un sentido amplio, en las personas, en las instituciones y en la sociedad.

## INTRODUCCIÓN

El problema del sinhogarismo ha experimentado, en términos estadísticos, un crecimiento sostenido durante los últimos años en diferentes partes del mundo. En el caso de la Unión Europea, se estima que en 2023 había un mínimo de 895.000 personas que dormían en la calle, se alojaban en refugios nocturnos, o bien en alojamientos temporales, lo que muestra un aumento en la mayoría de los países miembro respecto a años anteriores^1^. En España, el Instituto Nacional de Estadística informó que, en 2022, más de 28.000 personas sin hogar eran atendidas en centros asistenciales, lo que supone un aumento del 24% en relación con el 2012^2^. Este número representa únicamente a las personas atendidas en dichos dispositivos, con lo cual se estima que la cifra total es aún mayor. Para el caso de Barcelona, contexto en el que se desarrolla la presente investigación, los datos de 2022 apuntan a la existencia de 4.197 personas durmiendo en la calle, en asentamientos o en recursos del ayuntamiento, lo que supone un 98% más que el primer informe de 2008^3^.

En todos los casos se trata de personas que se enfrentan a diario a la vulneración de sus derechos fundamentales, quedando excluidas total o parcialmente de la posibilidad de ver cubiertas sus necesidades básicas: disponer de vivienda, de alimentación, de educación y de buena salud, entre otras necesidades imprescindibles para una vida digna. De esta forma, el acceso a bienes y servicios básicos se ve obstruido por su condición de extrema pobreza -falta de recursos económicos para adquirirlos-, pero también por la construcción social que se genera alrededor de su existencia al ser socialmente marcadas por el estigma, la discriminación y el aislamiento^4^^,^^5^^,^^6^. Al mismo tiempo, la experiencia del sinhogarismo está asociada a procesos de fuerte estigmatización que, en muchas ocasiones, tienen su reflejo en el trato que las personas reciben del equipo profesional y del propio sistema de atención en salud. De este modo, aunque España cuente con servicios de salud universales, públicos y gratuitos, se despliegan otro tipo de barreras no económicas que dificultan el acceso a los dispositivos sanitarios, generando incluso distancia y rechazo^7^^,^^8^^,^^9^^,^^10^. Esta situación retroalimenta las condiciones de precariedad, las dificultades en las condiciones de salud y el aumento del riesgo de muerte^11^^,^^12^^,^^13^^,^^14^.

El *Housing First* es una intervención social de larga trayectoria a nivel internacional, pero de incipiente desarrollo en el contexto español, que propone la entrada a una vivienda sin condiciones previas a personas con largas trayectorias de vida en la calle, que sufren trastornos mentales graves y/o adicciones. Este artículo tiene como objetivo analizar las experiencias que las personas participantes en el programa *Primer la Llar* tuvieron como usuarias del sistema sanitario, y con las personas profesionales que las atendieron. Se pretende de esta manera identificar los efectos que el ingreso en el programa, la disponibilidad de una vivienda y el soporte de los técnicos y las técnicas del *Primer la Llar* tuvieron en el proceso de cuidado y mejora de su salud, así como explorar la influencia que dicha circunstancia tuvo en los posicionamientos de los y las profesionales de la salud que las atendieron. Asimismo, se analizan las estrategias legas puestas en marcha por las propias personas en el ejercicio de su cuidado y mejora de su nivel de salud. Con todo, este estudio ofrece resultados de una experiencia en el sur de Europa, un contexto distinto al que generalmente son evaluados los programas *Housing First*, lo cual enriquece la disponibilidad de evidencia del modelo en nuevos territorios. 

### Modelo *Housing First* y su aplicación en Barcelona: Programa *Primer la Llar*

El modelo *Housing First* es un programa que ha generado prácticas innovadoras de atención a personas con largas trayectorias de sinhogarismo, trastornos mentales graves y/o adicciones, para quienes el circuito asistencial habitual no había logrado ofrecer una respuesta adecuada^15^. Comienza facilitando una vivienda de forma permanente e independiente, y la considera un derecho básico y facilitador de la recuperación. A diferencia del modelo tradicional de Escala o *Treatment First*, la entrada a la vivienda no está condicionada a un tratamiento específico, y la persona decide qué pasos dar para favorecer su bienestar y su incorporación comunitaria^16^^,^^17^. Las personas tienen un equipo de apoyo y sobre este aspecto existen dos tipos de abordaje. Por un lado, el *tratamiento asertivo comunitario* (TAC) para los casos de personas con trastornos mentales graves, con un equipo multidisciplinar que se reúne diariamente para evaluar los riesgos y las necesidades, y que cubre las 24 horas, los siete días de la semana^18^^,^^19^. Por el otro, el modelo de gestión intensiva de casos (GIC) para las personas con necesidades de tratamiento menos frecuentes, visitas domiciliarias una vez por semana y la utilización de servicios comunitarios para tratamientos psiquiátricos o médicos^20^. Desde la experiencia pionera de *Pathways to Housing*, liderada por Sam Tsemberis en Nueva York en la década de 1990, el modelo *Housing First* se ha extendido^21^^,^^22^ y se ha evaluado desde múltiples marcos teóricos y metodológicos, principalmente en los EEUU, Canadá, Australia y también en algunos países de Europa^17^^,^^18^^,^^19^^,^^23^^,^^24^^,^^25^^,^^26^^,^^27^^,^^28^^,^^29^.

En materia de vivienda, España se caracteriza por ser un país en el que su acceso presenta fuertes dificultades para una parte importante de la población. Si bien cuenta con un amplio parque residencial, este no responde a las necesidades de los colectivos con menos ingresos, especialmente aquellos que no pueden acceder a una vivienda de alquiler en el mercado inmobiliario y que requieren de viviendas sociales^30^. Con un índice del 2,5% de vivienda social respecto a las viviendas principales, España se posiciona como uno de los países con menor registro de vivienda social dentro de la Unión Europea (UE), quedando muy por debajo del promedio europeo del 9,3%^31^. A su vez, en comparación con otros países de la región, España es el territorio de la Unión Europea donde las personas que alquilan destinan mayor porcentaje de sus ingresos a sufragar el costo de la vivienda^32^. En este contexto, el Ayuntamiento de Barcelona se convierte en 2015 en la primera administración pública española en poner en marcha el modelo *Housing First* con el programa piloto *Primer la Llar*. El programa facilitó viviendas con apoyo a 50 personas en situación de sinhogarismo^33^. Para ello, se seleccionó a personas con largas trayectorias de vida en la calle, trastornos mentales y/o adicciones, autonomía funcional para la vida diaria y disponibilidad de ingresos o capacidad de tenerlos. A partir de esta experiencia se realizó el seguimiento y evaluación mediante un abordaje cualitativo y longitudinal, del cual surge el presente artículo.

### Condiciones de salud en trayectorias de sinhogarismo y el rol del estigma en el acceso al sistema sanitario

Los factores sociales suponen un determinante de la salud, que condiciona también las posibles formas de búsqueda de apoyo social en el ámbito formal y no formal^34^^,^^35^. Al respecto, este estudio adopta un concepto ampliado de salud^36^^,^^37^ que incluye la dimensión biológica, pero también los factores sociales, culturales, psicológicos y políticos que influyen en el estado de salud de las personas, evitando así un reduccionismo más propio del modelo biomédico. Este concepto de salud no es unívoco ni universal, “como todas las prácticas lingüísticas, la salud es metafórica: absorbe y expresa una variedad de significados que se encuentran en toda la cultura”^38^. Esto induce a pensar la pertinencia de realizar estudios cualitativos, con abordajes centrados en las personas y sus contextos para proponer un análisis respetuoso que asuma una comprensión amplia de los sufrimientos vividos y los consiguientes itinerarios de atención por los que han transitado las personas. Así mismo, el trabajo con las narrativas nos ha llevado a adoptar el análisis de las problemáticas de salud a partir del concepto de malestar^39^ por el que hemos podido aproximarnos a las estrategias legas con las que las personas participantes han afrontado las problemáticas de salud.

Las personas con largas trayectorias de sinhogarismo suelen disponer de una salud física y emocional precaria. La tasa de morbilidad y mortalidad es generalmente más alta que para el resto de la población, especialmente en jóvenes y mujeres^8^^,^^10^^,^^40^^,^^41^. A su vez, el hecho de no tener una vivienda aumenta los síntomas de los problemas de salud mental y el consumo de drogas^42^^,^^43^ y, paradójicamente, pese a requerir más atención sanitaria, suelen tener menos acceso a la atención^44^^,^^45^. En el caso de España, diversos estudios corroboran este hecho que se produce en distintas ciudades y centros de atención^14^^,^^46^^,^^47^^,^^48^. Los abordajes de *Housing First* suelen arrojar resultados positivos sobre las condiciones de salud de las personas que acceden a estos programas. Al respecto, diversos estudios muestran el papel crucial de una vivienda adecuada para el bienestar emocional de las personas^49^^,^^53^, así como la disponibilidad de un espacio de vida propio puede mejorar la efectividad de los tratamientos, al permitir seguir hábitos sin el estrés causado por el sinhogarismo^16^. Se argumenta que, contar con una vivienda facilita el proceso de recuperación, ya que la vivienda es el lugar en el que la persona puede ser ella misma al margen del mundo exterior, donde se crea la identidad, las rutinas y se desarrolla la vida cotidiana^54^^,^^55^^,^^56^. Sin embargo, desde este enfoque, la recuperación no es un objetivo fijo, común para todas las personas, sino un horizonte adaptado a la subjetividad y a las características particulares. Siguiendo a Leamy *et al*.^57^ la recuperación es un camino, está configurada por dimensiones y la conforman diferentes fases. Es un concepto complejo que es útil siempre que no imponga unas características determinadas ni unas expectativas irreales. Palimaru *et al*.^58^ apuntan que el modelo *Housing First*, en comparación con otros abordajes de sinhogarismo, resulta más efectivo en cuanto a la prestación, coordinación y utilización de servicios de salud, pero que, sin embargo,

…los programas *Housing First* y no *Housing First* comparten una serie de desafíos sistémicos, incluida la naturaleza compleja y lenta de trabajar con clientes de alta gravedad, servicios de salud mental insuficientes, rotación de administradores de casos y escasez de personal, que pueden socavar algunos de los beneficios del enfoque *Housing First*.^58^

El estigma, entendido como situación individual que inhabilita una plena aceptación social^59^, es un elemento esencial para entender los procesos de recuperación, los cuales presentan mayor complejidad para las personas que se encuentran en situaciones de extrema vulnerabilidad, ya sea por pobreza, problemas de salud mental^60^^,^^61^, adicciones^62^ y/o diversidad funcional, entre otras, generando una estigmatización múltiple^63^. 

La literatura indica que hay una tendencia a situar a estas personas dentro de una ciudadanía pasiva, al verlas como personas dependientes, tanto económica como socialmente del Estado, despojándolas de su poder de agencia; mientras que aquellas personas que son independientes suelen ser vistas como ciudadanas activas y plenas^64^. Las raíces de estos comportamientos discriminatorios se encuentran en los parámetros estructurales de las sociedades que definen las características de la normalidad y excluyen a quienes se alejan de las mismas. Las relaciones de poder entre los distintos actores de una comunidad se vuelven fundamentales para ejercer estos comportamientos^60^. 

Link y Phelan^65^ señalan la existencia de un *poder del estigma,* dado que:

…los estigmatizadores tienen fuertes motivaciones para mantener a las personas reprimidas, dentro o fuera, y que la mejor manera de lograr estos objetivos es a través de procesos de estigmatización que son indirectos, ampliamente efectivos y ocultos en circunstancias culturales que se dan por sentado.^65^

Los mecanismos estigmatizadores operan ampliamente en las personas que atraviesan situaciones de sinhogarismo^4^^,^^5^^,^^6^ y generan consecuencias en múltiples sentidos como mayor aislamiento, dificultad en los vínculos sociales y con las instituciones^9^^,^^65^. Belcher y DeForge^4^ señalan, por ejemplo, el rechazo que produce en una comunidad el anuncio de la construcción de un albergue para personas sin hogar, ya que quienes habitan ese barrio no quieren tener esta población cerca, identificada como problemática y no deseable. De este modo, muchas personas sin hogar reconocen sentir el estigma tanto en la sociedad en general como dentro de los dispositivos de atención médica^66^^,^^67^^,^^69^.

Múltiples estudios abordan los vínculos existentes entre personas sin hogar, con problemas de salud mental y/o adicciones y sus experiencias en el sistema de salud, identificando situaciones estigmatizantes y las consecuencias que ello genera en las trayectorias de esta población^8^^,^^10^^,^^13^^,^^69^. Algunas de las actitudes con las que se encuentran por parte de los y las profesionales médicos son: la infantilización; la falta de respeto e interés^60^; la creencia de que se acercan a emergencias con el objetivo de obtener prescripciones para narcóticos, basada en el estereotipo de que son personas adictas^70^; y la presencia de comentarios culpabilizantes sobre la falta de cuidado de su salud^71^, entre otras. Además, también se encuentran con una barrera previa que es la del personal administrativo o de seguridad, que puede también ejercer prejuicios y actos de discriminación^11^ y, en algunos casos, se añaden otras características de las personas que hacen que se profundice esta situación como, por ejemplo, el caso de las personas sin hogar migradas que acceden aún con mayores dificultades y desigualdad al sistema de salud^14^. Estas experiencias de discriminación no solo generan un acceso deficiente a los tratamientos, sino que en sí mismas influyen en la salud a través del estrés que generan en las personas que se exponen a ello, que puede causar enfermedades físicas y psíquicas^12^.

La otra cara de la misma realidad es el papel ampliamente favorecedor que pueden tener los vínculos con el equipo de salud en los procesos de recuperación^58^^,^^72^^,^^73^^,^^74^. Padgett *et al*.^9^ analizan las condiciones que facilitan o limitan los tratamientos de personas sin hogar que tienen problemas de salud mental severos o consumo de drogas e identifican que uno de los aspectos facilitadores son los “actos de bondad” por parte del personal médico, en los que muestran humanidad y calidez, los cuales rompen con la norma de los encuentros de rutina y deshumanizantes con los que suelen encontrarse. En este sentido, Fang *et al*.^5^ utilizan el concepto de “humildad cultural” bajo el cual proponen que el personal médico, a través de un ejercicio de autorreflexión, sea consciente del poder que tiene en estos vínculos y que eso les permita empatizar con las creencias y experiencias de las personas que reciben para adaptarse a las particularidades, entendiendo que una única perspectiva cultural no es aplicable a todas las personas.

## METODOLOGÍA

### Diseño, población y muestra

Esta investigación utilizó una metodología cualitativa^75^, desde una perspectiva interpretativa y con un diseño longitudinal. La adopción del enfoque narrativo permitió hacer emerger elementos de complejidad para comprender las experiencias de las personas participantes en el programa, mientras el uso de la perspectiva longitudinal posibilitó observar la aplicación del programa en la vida de las personas a lo largo del tiempo, siguiendo las investigaciones realizadas a escala internacional^26^^,^^76^^,^^77^^,^^78^. La investigación se estructuró en tres fases entre 2016 y 2020. En cada una se realizaron entrevistas semiestructuradas a los participantes^79^, que han sido diseñadas y llevadas a cabo por un equipo de investigación compuesto por profesores y profesoras de la Unitat de Formació i Recerca - Escola de Treball Social de la Universitat de Barcelona, responsables de la autoría de este artículo. 

El programa Primer la Llar contó con una población de 50 personas, de las cuales 20 formaron parte de la muestra continua y final de esta investigación (n=20), es decir, el 40% de la población. En relación con otras investigaciones de características similares, se considera una muestra elevada^80^. La selección muestral fue de carácter intencional y se mantuvo el 90% de la muestra original. Se dispuso de una reserva de tres personas, en caso de producirse alguna baja durante la investigación. El promedio de edad de las personas participantes fue de 55 años. La Tabla 1 muestra las características de la muestra final, según género, origen, diagnóstico inicial, cronicidad (definida por el U.S. Department of Housing and Urban Development^*81*^ y estado vivil)

**Tabla 1:** Características de la muestra (n=20). Barcelona, 2016 y 2020.

Variables	n	%
Género		
Hombres	16	76
Mujeres	4	69
Origen		
Autóctonos/as	14	70
Migrantes comunitarios	1	5
Migrantes extracomunitarios	5	25
Diagnóstico inicial		
Adicción	6	30
Patología Dual	3	15
Trastorno mental	7	35
Otros/desconocido	4	20
Cronicidad*		
1 a 5 años	8	40
6 a 10 años	7	35
Más de 10 años	5	25
Estado civil		
Separado/a	10	50
Soltero/a	6	30
Divorciado/a	3	15
Casado/a	1	5

Fuente: Elaboración propia. * Chronically homeless definida por el U.S. Department of Housing and Urban Development, 2015^81^.

### Recolección y análisis de datos

El trabajo de campo se ha desarrollado en tres fases, con entrevistas al inicio, a los 24 y a los 40 meses, diseñando y adaptando los guiones a cada una de las fases de la investigación. La primera fase, realizada de mayo de 2016 a junio de 2017, se centró en conocer las historias de las personas al entrar al programa y sus trayectorias de vida durante ese periodo previo. Ello implicó conocer los procesos por los que habían transitado a nivel vital, incluyendo aquellas experiencias que les habían generado mayor sufrimiento, las trayectorias habitacionales seguidas y los contactos previos con recursos especializados de atención social. La segunda fase, realizada de mayo de 2018 a enero de 2019, se inició cuando las personas ya llevaban dos años en el programa y tuvo como objetivo conocer el proceso de adaptación a la nueva vivienda y el curso de su recuperación personal y social, experimentado a partir de la entrada. En la tercera y última fase, realizada entre septiembre de 2019 y enero de 2020, el trabajo se centró en conocer los cambios experimentados después de permanecer tres años en el programa, así como la autoevaluación que las propias personas participantes habían hecho de esos cambios y de las repercusiones en su estado actual. La duración de las entrevistas osciló entre 2 y 5 horas de duración, dependiendo de si eran una o dos sesiones, y se realizaron mayoritariamente en espacios públicos (cafeterías, centros municipales, espacios abiertos) y en algunos casos en los propios domicilios, según la disponibilidad y comodidad expresada por las personas participantes. 

El trabajo de campo se adaptó a las necesidades de las personas entrevistadas. Al inicio, el contacto se estableció mediante el equipo de profesionales del programa. Esto permitió valorar conjuntamente el mejor momento para hacer la entrevista. En las siguientes etapas, el contacto fue directo con las personas a entrevistar. Las grabaciones fueron transcritas de forma literal y se elaboraron códigos a partir de las preguntas y objetivos del estudio que se ajustaron a las temáticas incorporadas en cada fase, se añadieron los códigos emergentes consensuados por el equipo de investigación, y en cada etapa se realizó revisión por pares. El análisis se realizó con Atlas.ti 8.

### Aspectos éticos

Esta investigación siguió el *Código ético de integridad y buenas prácticas en investigación* de la Universitat de Barcelona, de 2019. Cada persona aceptó voluntariamente participar en la investigación, con el compromiso de utilizar los datos solo para los fines de la investigación según la Ley orgánica 15/1999 y la Ley de Protección de datos personales y garantía de los derechos digitales - Ley Orgánica 3/2018 de 5 de diciembre. Se informó verbalmente y por escrito a cada persona entrevistada sobre los objetivos de la investigación, se les facilitó y firmaron un consentimiento libre e informado y se les entregó una copia de la transcripción de las entrevistas en cada una de las fases. Se garantizó el anonimato, utilizando pseudónimos en el presente artículo, y la confidencialidad de los datos.

## RESULTADOS

### Principales efectos del programa *Primer la Llar* en las trayectorias de salud

La primera fase de la investigación reveló que las personas participantes padecían importantes problemas de salud física, al identificarse enfermedades con diferentes niveles de gravedad. Dentro de las más leves se mencionaron los problemas bucodentales, de oído, de vista, diabetes, colesterol y afectaciones a la tensión. Las más graves fueron cáncer, lesiones de espalda y hepatitis C. Por otra parte, de acuerdo con los relatos, estas enfermedades se veían agravadas por el propio itinerario vital, la precariedad en las condiciones de vida, el consumo de alcohol, la falta de acceso a recursos sanitarios y, en otros casos, por el mismo proceso de envejecimiento, como ocurre con la lumbalgia o las enfermedades neurodegenerativas. En la segunda y tercera fase de la investigación pudimos evidenciar que, para la gran mayoría de las personas entrevistadas el cambio de situación habitacional había supuesto importantes modificaciones en su estado de salud.

Claro que me ayuda. No es lo mismo estar en la calle que estar acogido en un techo. Si estuviera en la calle, no sé ni cómo estaría. (Miguel Ángel, entrevista fase 2)

Mejor que antes. Antes bebía mucho, ahora no. Cuando estaba en la calle bebía mucho. Aunque ya me dijo el médico, tomaba el Antabús, lo dejaba de tomar… bueno, todo muy mal. La diabetes igual. Con diabetes, bebía, fumaba… un desastre. Ahora mejor, desde que tengo el piso mejor, está más controlado todo. (Omar, entrevista fase 2)

Hombre, el piso a mí me ha ayudado mucho. Si quiero estirarme, me estiro. Si me duele algo me siento. Me ayuda mucho. Es la base de toda la salud. Si estás en el albergue y quieres echar la siesta no te dejan. Te duele la cabeza, no te dejan; tienes fiebre, no te dejan. Muchas cosas... que he visto a gente que lo ha pasado mal. Pero aquí en casa puedes hacer tu vida. […] medicina, tú pones tu salud. La casa es la base. (Youssef, entrevista fase 3)

Una cuestión que emergía en los relatos eran los problemas de salud bucodental y las repercusiones físicas y mentales que habían originado en las personas en el contexto de una alimentación deficiente e inadecuada, lo que los había llevado a sufrir infecciones y complicaciones digestivas, entre otros problemas de carácter físico. Al mismo tiempo, y en los casos más extremos, se percibía como un problema de salud que, exterioriza y hace aún más visible la pobreza, operando en un plano simbólico que afianza el estereotipo de la persona sinhogar y que contribuye al aislamiento social, la baja autoestima y los problemas de comunicación.

Porque yo por dentro pensaba, ya que estoy aquí, ya que he dado el paso, por lo menos hacer vida social, cuando ya esté bien del todo, cuando tenga la dentadura, cuando ya... Porque yo sigo siendo yonqui de dientes, ¿sabes? (Alfonso, entrevista fase 3)

En cuanto a experiencias de sufrimiento mental, se observaron en las entrevistas reticencias a hablar de ellas, especialmente si se planteaban desde la terminología médica. Sin embargo, en los relatos se percibía un reconocimiento implícito del posible sufrimiento mental que podía darse como resultado de la vida en la calle.

Desde que estoy en la calle tirada... ahí es cuando empezó mi cabeza a funcionar de otra manera. Como no salían las cosas como yo quería, me cerraba, me bloqueaba. No encontraba otra salida. (Maria José, entrevista fase 2)

En algunos casos, como la entrevista a María José, se evidencia un importante trasfondo de dolor y sufrimiento, independientemente de la existencia o no de un diagnóstico clínico. Así, en los relatos se mencionan los años de sufrimiento, de angustia, de “haber pasado mucho”, de “sentir un malestar muy grande dentro” y de “no querer remover”, reconociendo la experiencia de un proceso personal de sufrimiento frente al que se prefiere “vivir el presente”, eludiendo el afrontamiento del posible malestar vivido y sus causas. Por otra parte, se reconoce que el acceso a la vivienda les ha proporcionado una situación de mayor tranquilidad, seguridad, estabilidad y ganancia de autoestima que ha influido positivamente en su bienestar físico y psíquico.

Pues de valorar más las cosas, sentirme bien conmigo mismo…, de poder hacer lo que quiera, ¿no? Es lo que te he dicho antes. No dependes de nadie, de que nadie te esté diciendo que tengas que hacer esto, o lo otro… y que puedas ser autónomo. (Miguel Ángel, entrevista fase 2)

Por otro lado, en la tercera fase de la investigación se observa que la vivienda y las propias limitaciones temporales que impone el programa plantean un panorama de incertidumbres con las que debe convivir. De esta forma, emerge la angustia y el malestar frente a la posible situación habitacional futura. Una situación que termina siendo una de las mayores fuentes de malestar relatadas por las personas entrevistadas, con especiales consecuencias sobre la salud mental.

Yo vivo tranquila, pero no sé mañana qué va a pasar. (María, entrevista fase 3)

### Acceso al sistema de salud, ¿cambio o continuidad?

En la primera fase de la investigación se obtuvieron relatos sobre el funcionamiento del sistema de salud para la población sin hogar -es decir, sus experiencias previas a ingresar al programa- lo que permitió identificar diferentes problemas: carencia de coordinación entre los hospitales y los equipamientos de la red de servicios sociales, exigencias administrativas y largos tiempos de espera para acceder a los dispositivos. La valoración más crítica sobre el acceso o aspectos del funcionamiento de algunos dispositivos de salud la realizaron, sobre todo, personas consumidoras de drogas. Por ejemplo, se cuestionaba la exigencia de estar empadronado en un domicilio fijo para poder acceder a un tratamiento de metadona. Este criterio administrativo, que puede tener una lógica de organización de los servicios de acuerdo con la población usuaria de cada territorio de la ciudad, a su vez puede actuar como una barrera disuasoria hacia el acceso al tratamiento para aquellas personas que no pueden cumplir este requisito. También se cuestionaron los horarios de algunos servicios, por ejemplo, las salas de venopunción, dispositivos que responden a una política y a programas de reducción de daños, ya que las personas consumidoras, tal y como se puso en evidencia en los relatos, pueden no seguir necesariamente los mismos tiempos y pautas en sus formas de consumo.

En la segunda y tercera fase de la investigación encontramos que, una vez disponían de una vivienda en el marco del programa *Primer la Llar*, las personas participantes continuaban manifestando dificultades en el acceso a la salud debido a ciertos funcionamientos institucionales, como los cambios o las rotaciones de profesionales que dificultan la creación de posibles vínculos estables y pueden generar desconfianzas.

Sí, a la hora de [ir al] psiquiatra, que de psiquiatra me han cambiado tres veces en lo que llevo allí… Así que les cuento la misma historia cada vez... (Miguel Ángel, entrevista fase 2)

También se evidenció cómo el propio estigma asociado a los trastornos mentales puede representar una barrera a la hora de asistir a la consulta de un profesional de salud mental, en tanto no acababa de entenderse la utilidad del tratamiento y se convierte en una propuesta que puede generar rechazo.

Yo iba a una psicóloga, pero lo dejé cuando entré aquí́. Lo más gordo me quieres meter... Yo iba a una que el objetivo era saber si era puta, si fumaba, etc., después a otro... no me van a liar otra vez con esta gente. No me liaréis otra vez con esta gente. No quiero saber nada de ellos. No iré́, no me liaréis otra vez con esta gente. No iré́, no volveré́ atrás. (María, entrevista fase 2)

Sí, pero ahora no estoy preparada tampoco para ir otra vez. Porque sé que cuando voy me van a hacer recordar, me hacen preguntas y ahora no… digo, lo vamos a dejar así. A lo mejor más para adelante sí que voy a ir porque me vendrá todo de golpe y tanta felicidad tampoco es buena. Yo lo sé. Todo esto bueno… luego tiene sus altibajos. (María José, entrevista fase 3)

Sin embargo, también surgen experiencias positivas que muestran la utilidad práctica de un espacio psicoterapéutico, y cómo la generación de vínculos de confianza entre las partes puede favorecer el proceso de recuperación de las personas atendidas.

Sí, estoy yendo a una psiquiatra y me va bastante bien porque me desahogo mucho. O sea, digo eso que a lo mejor en la calle no me atrevo. (David, entrevista fase 3)

En diferentes relatos se evidencia cómo la vivienda favoreció la asistencia a las visitas médicas y el seguimiento de los tratamientos propuestos. De hecho, la totalidad de las personas entrevistadas hablan del Centro de Atención Primaria (CAP) como el dispositivo sanitario de referencia y del hospital como el servicio utilizado en caso de urgencia.

He sufrido en la calle muchos años y para tener un piso, tu llave, tu casa, hacer lo que quieras, salir cuando quieras, entrar, es una lotería. Para mí es una alegría grande. Si estás en la calle, te empeoras más y no tienes tiempo de seguir las medicinas, la medicación, no tienes ganas de nada... tienes que estar como un animal tirado. No es como en la casa, que limpias, encuentras ánimos, te da fuerzas para hacer cosas. (Said, entrevista fase 2)

El contacto asiduo con el equipo médico del CAP y con la farmacia del barrio les han permitido un mayor acceso a fármacos como medida terapéutica, lo que es valorado por la mayoría de las personas entrevistadas como forma de mitigar el dolor físico y mental. Así mismo, la mayoría de las personas participantes han percibido un beneficio directo al sentir una importante mejora en el control de determinados síntomas, mientras que indirectamente parece asumirse como una fórmula que permite evitar un posible afrontamiento psicoterapéutico, y el consiguiente abordaje de episodios de intenso dolor psíquico.

A su vez, se observó en algunos relatos una evolución en la relación entre las personas participantes del programa *Primer la Llar* y el personal sanitario, visibilizando un aumento en la calidad de los vínculos de confianza y de acompañamiento, que repercute positivamente en el estado de salud de las personas.

Todos han sido muy claros: han hablado conmigo, me han expuesto las cosas sin chillar, que es como me gustan las cosas. Me las han expuesto muy bien, tanto la neumóloga como el señor Tomás, el de cabecera. Me han tratado como lo que es todo el mundo, como una persona, no como ganado. (Fermín, entrevista fase 2)

De hecho, las entrevistas señalan que con algunos miembros del equipo profesional se produjo un contacto más estrecho y continuado; por ejemplo, el personal de enfermería es valorado como más accesible en la práctica y son con quienes suelen mantener visitas más continuadas, o también con la persona que atiende en la farmacia de referencia, alguien a quien se puede acceder con relativa asiduidad y de forma no protocolizada y que, a pesar de no contar muchas veces con formación específica en el ámbito social, puede llegar a formar parte de una estructura de cuidado que será referencia.

Sin embargo, aunque el hecho de disponer de una vivienda las aproxima a llevar una vida socialmente aceptada y a tener más capacidad para reclamar sus derechos, en algunos casos perciben que llevan la huella del estigma social de la persona que ha vivido en la calle. De esta manera consideran que les resulta muy complicado integrarse en la sociedad en la que viven cuando han estado fuera.

Yo para ellos continúo siendo uno sin techo, como si dijéramos, con ellos soy un usuario con excedencia. Esto lo he visto siempre. (Alfonso, entrevista fase 3)

Asimismo, perciben que continúan ubicados al margen, y que muchas veces reciben un tratamiento de manera diferenciada respecto al resto de la población, sintiendo la segregación y exclusión de ciertos espacios, en el que los prejuicios y los discursos estigmatizantes existentes siguen colocándoles etiquetas que justifican prácticas y acciones discriminatorias hacia ellos y ellas.

Miguel Ángel: Sin mirar en tu pasado, que no por el pasado que hayas tenido te cataloguen ya como si fueses un bicho raro.

Entrevistadora: Eso lo has sentido algunas veces y piensas que sería mejor si…

Miguel Ángel: Lo sentí allí en [nombre de asociación dedicada a la salud mental]. (Miguel Ángel, entrevista fase 3)

### Más allá de las instituciones: estrategias propias del cuidado de la salud

Las narrativas mostraron también que las personas participantes del programa habían desarrollado estrategias vinculadas a cierto tipo de terapias alternativas, a autogestionarse el tratamiento y a posicionarse frente al personal sanitario. Se trata de habilidades aprendidas y elaboradas a partir de su propia experiencia vital. Por ejemplo, recurrían a la meditación o a la práctica del budismo como medios de relajación y espiritualidad, llamaban por teléfono a la familia para calmarse o incluso asumían el delirio como parte del funcionamiento personal. Algunas estrategias estaban relacionadas con equipamientos comunitarios y de proximidad, como apuntarse al gimnasio para obligarse a salir de casa y afrontar la fobia social, y otras se relacionaban con el uso de la casa como, por ejemplo, dormir más, o el uso de remedios naturales que antes no podían ser preparados en la calle.

No sé, me he apuntado al gimnasio, porque, está claro, es que no salía de casa. Aquí encerrada me sentía segura, porque me cogía fobia. Es que cuando digo fobia... que no tenía ganas de ver gente. Y ahora me cuesta también. Y coger el metro, pero ahora, como lo tengo que coger cada día para ir al Clínico... Entonces me apunté al gimnasio y me va bien. (Silvia, entrevista fase 2)

Las experiencias de sufrimiento han dado paso a un mayor autoconocimiento sobre los efectos de los tratamientos y una mayor seguridad y agencia frente a las propuestas médicas. De esta forma, en la última fase de la investigación, se han observado también prácticas de autogestión de la medicación.

Pues no sé, es que según como me sienta yo también. Es que hay días que, si me tiro días sin tomarla, el cuerpo mismo te la pide, te pide esas sustancias, eso lleva morfina y claro eso lo necesita el cuerpo, es una sustancia que necesita el cuerpo. Es como los parches de morfina que ponen a la gente fumadora. No fumadoras no, parches de morfina que son para gente que tiene dolores. Por eso yo la necesito, pero no la necesito diariamente, cuando yo me siento mal, me siento con bostezo me la tomo, hoy por ejemplo me la he tenido que tomar, esta mañana me la he tomado, pero a lo mejor mañana no me la tomo. (Miguel Ángel, entrevista fase 3)

En relación con el apoyo social, se ha evidenciado en algunos casos cómo la persona de referencia del *Primer la Llar* se convierte en la figura acompañante o de apoyo para las visitas al sistema de salud, escogida así por las personas participantes como alguien de confianza, con la intención de generar una mayor escucha por parte del personal sanitario hacia su situación.

Me gusta mucho ir con la referente 32, porque siempre ella me recuerda algo. Y si no, lo dice, porque ella tiene carta blanca para hablar lo que quiera. Todos los que me acompañan a los médicos, todos, tienen carta blanca por, si se me escapa algo, entonces decirlo ellos. (Fermín, entrevista fase 2)

Asimismo, la vivienda se constituye, en algunos casos, en un espacio de cuidados y/o de autoprotección en el que refugiarse en caso de sentir malestar, lo que les ha permitido tener un mayor control en actuaciones relacionadas con el autocuidado y las tareas del hogar. Al respecto, en las narrativas se destaca la importancia de ser autosuficientes a la hora de realizar estas tareas, y que su autocuidado no solo haya sido positivo para su salud física, sino también para su estado de ánimo, señalando que los ha llevado a mejorar su autoestima. Además, una gran cantidad de participantes ha considerado que disponer de una vivienda les ha ayudado a mejorar sus hábitos de alimentación y su bienestar: han mejorado su peso y forma física, han encontrado mejoras en algunas enfermedades (colesterol, diabetes) y la posibilidad de decidir lo que comen en horarios adecuados.

A lo mejor, en la calle comía una vez al día, y aquí como cuatro o cinco. Ha cambiado todo. (María José, entrevista fase 2)

Otro de los hábitos vinculados a la salud y recuperados a partir de disponer de una vivienda es la higiene y la voluntad de mejorar su imagen personal. Los medios con los que se proponen conseguir esta mejora son el ejercicio físico, la forma de vestir y los tratamientos bucodentales.

Cuando llegas a un piso como este, es que solamente ver la casa y verte aquí́ te dan las ganas de poder ducharte. (Manuel, entrevista fase 3)

Estas estrategias también han sido utilizadas para el afrontamiento de adicciones en el caso de las personas participantes que manifestaron haber consumido algún tóxico (principalmente alcohol y marihuana, pero también tabaco). El deseo de reducir o suprimir este consumo ha generado la aparición progresiva de fórmulas de afrontamiento, algunas a partir de las pautas del equipo de profesionales, como la asistencia al Centro de Atención y Seguimiento (CAS) a las drogodependencias o la ingesta de medicación específica (Antabús, en caso del alcohol). Pero, en otros casos, se ha llevado a cabo a partir de la modificación de hábitos, como el cambio de amistades, relaciones, o la modificación de ciertos consumos en favor de otros menos nocivos. En este sentido, se evidencia que disponer de una vivienda permite también cierta limitación y control, y la instauración de nuevos hábitos de vida más saludables. Sin embargo, algunas personas mencionan que les cuesta abandonar rutinas de la vida en la calle, como los malos hábitos alimentarios y que, por razones económicas, continúan recurriendo a comedores sociales o tickets restaurantes, en vez de comer en casa y de forma más saludable.

## DISCUSIÓN

Las narrativas de las personas participantes en el programa *Primer la Llar* nos han servido para aproximarnos a las experiencias vividas respecto a su estado de salud y a la forma en la que acceden a los cuidados a partir de disponer de una vivienda, a la vez que nos han permitido acercarnos a sus percepciones sobre la relación con los equipos profesionales del sistema sanitario y el desarrollo de estrategias propias para su cuidado personal, más allá de las instituciones.

En primer lugar, este análisis confirma que los procesos de recuperación son complejos y diversos^57^, así como las personas participantes y sus trayectorias, por lo cual se observa que tanto sus estados de salud como las formas de afrontar el contacto con profesionales, tratamientos, terapias y el sistema sanitario también lo son. Lejos de pretender encontrar un patrón uniforme o una única respuesta, la metodología cualitativa e interpretativa nos permite profundizar en las múltiples experiencias vividas.

Respecto a su estado de salud se identifican una serie de dolencias y enfermedades previas al ingreso al programa, similares a las indicadas por otros estudios y que prevalecen en personas que atraviesan períodos de sinhogarismo^14^^,^^40^^,^^41^^,^^42^^,^^43^^,^^46^^,^^47^^,^^48^. Explican que, en la calle, el control de los síntomas que se sufrían era escaso o bien era objeto de un seguimiento muy esporádico. Sobre la base de sus experiencias se observa que la carencia de una vivienda de referencia, la posible fragilidad de la red social y la dificultad de acceso a la información han sido claves en esta situación de desventaja. Observando el paso del tiempo en la investigación y los procesos que se fueron sucediendo se identifica que, en algunos casos, la vivienda se irá constituyendo progresivamente como espacio de vida, ayudando a controlar los efectos perniciosos que la misma calle o los dispositivos de asistencia previos han podido tener en la salud de las personas. A lo largo de estos cuatro años se ha visto afianzada la relación entre las personas participantes en el programa y el equipo profesional de los servicios sanitarios o el personal que se relaciona con este contexto, como puede ser la persona que atiende en la farmacia. En muchos casos el contacto ha sido cada vez más estrecho teniendo en cuenta el aumento de las pruebas y de los seguimientos periódicos de salud realizados.

Sin embargo, las dificultades que constituyen un obstáculo hacia la recuperación de las personas participantes en el programa son, en muchos casos, consecuencias de todo un itinerario vital. Este itinerario se ha caracterizado por la exclusión económica y por problemas de salud pero, además, por una cotidianidad diferente a la de la mayoría de las personas y una relación a menudo complicada con las instituciones que tienen por misión dar respuesta a las personas en situación de exclusión social. En este punto se incorpora la variable de la estigmatización, analizando puntualmente los efectos de esta actitud discriminatoria generada por parte de los equipos del sistema sanitario hacia las personas que están o han estado en situaciones de sinhogarismo y que también pueden presentar problemas de salud mental y/o adicciones. Esta acción dificulta aún más el acceso a las visitas médicas y constituye en sí misma una experiencia dolorosa. Esta relación, evidenciada en otros estudios^8^^,^^10^^,^^11^^,^^12^^,^^13^^,^^69^^,^^70^^,^^71^ fue identificada en algunas de las personas participantes en esta investigación, por momentos de manera explícita y en otros de manera más indirecta como, por ejemplo, cuando afirman ir a las consultas con una persona acompañante del programa porque así recibirán mejor atención. Los relatos de las personas participantes han mostrado estas experiencias también en relación con otras instituciones o espacios de socialización. Se evidencia que el estigma del sinhogarismo sigue siendo un obstáculo que dificulta el acceso a la plena ciudadanía e integración social.

Paralelamente a la identificación de las barreras materiales y simbólicas en el sistema de salud, se observan estrategias propias que desarrollan las personas participantes del programa para autogestionar diversos cuidados. Algunas de ellas ya las realizaban cuando no tenían un hogar y otras son prácticas nuevas que son posibles a partir de tener una vivienda, vinculadas generalmente a la consecución de una mayor autonomía. Las encontramos en los relatos que hablan sobre los modos para transitar días difíciles, situaciones angustiantes o dolores físicos y tienen que ver tanto con la gestión de medicamentos como con prácticas asociadas a la relajación, la espiritualidad y el cuidado en la alimentación e higiene. Si bien resulta ampliamente favorecedora la capacidad de utilizar herramientas propias para la gestión del malestar, el marco desde el cual se realiza el presente análisis no pretende interpretarlo como el desarrollo creativo de estrategias para adaptarse a una fuerte vulneración de derechos. Esta visión correría el riesgo de mantenernos en una postura que se centra en la responsabilidad individual, que encapsula el problema en aspectos de la persona y que disminuye el peso que realmente tiene la problemática, tanto del sinhogarismo como de la estigmatización, a nivel social, colectivo y político^60^. Resulta necesario que la capacidad de agencia particular de cada persona se extienda hacia lo grupal y colectivo, con el objetivo de que no quede en estrategias aisladas y frágiles. La mayoría de las intervenciones y actividades realizadas durante el programa *Primer la Llar* fueron de carácter individual y no se generaron espacios grupales para trabajar este aspecto. Para acercarse a la recuperación se precisa un contexto que acompañe y la facilite y, para ello, las intervenciones deben buscar también influir en ese contexto para generar otras condiciones de existencia^4^. En línea con la literatura presentada anteriormente, esto requiere trabajar una sensibilización colectiva y reconocer la existencia de dos procesos: uno propio y otro de la comunidad, donde se identifiquen las barreras culturales y la capacidad de respuesta de los servicios^5^.

Respecto a las limitaciones de la investigación que enmarca el presente artículo se apunta, en primer lugar, la particularidad de realizar trabajo de campo con personas que tienen trayectorias de sinhogarismo extensas y con múltiples experiencias de vulneración de derechos. En algunas ocasiones, ello comporta cierta desconfianza hacia la investigación, las personas que investigan, las entrevistas y el hecho de relatar, una vez más, sus historias de vida, muchas veces dolorosas. Esto puede reflejar algunas declinaciones en la participación, apareciendo el cansancio hacia las entrevistas, a no ser escuchado o escuchada, a la repetición y a la sobre observación. Se asume con ello la limitación de una mayor probabilidad de desgaste muestral que tienen las investigaciones longitudinales. En segundo lugar, se apunta que la muestra se ha visto condicionada por los tiempos de incorporación de las personas al programa, haciendo que se seleccione en mayor medida personas que ingresaron en un segundo momento. Finalmente, el carácter innovador del programa nos coloca en una experiencia de la cual tenemos muchas referencias teóricas, pero no prácticas.

## CONCLUSIONES

Este estudio evidencia que las personas con largas trayectorias de vida en la calle, que sufren trastornos mentales graves y/o adicciones pueden encontrar en un programa como *Primer la Llar* otras oportunidades de cuidado de su salud y de vinculación con el sistema sanitario. Así, el hecho de tener una vivienda les ha permitido realizar prácticas y hábitos de cuidado cotidianos básicos que se han ido instalando como rutinas saludables, y disponer de un espacio donde sentirse bien anímicamente. Sin embargo, en algunos relatos se ha identificado que el acceso a los servicios de salud continúa siendo limitado por actitudes discriminatorias, ya que cargan con el estigma social de la persona que ha vivido en la calle.

Frente a esta realidad se evidencia que, además de implementar estrategias para solucionar el problema del sinhogarismo enfocadas en las personas que atraviesan esta situación, resulta necesario repensar de qué manera las instituciones, las organizaciones y la comunidad en general se vinculan con esta población. Para ello, es preciso continuar reflexionando sobre la categoría de “normalidad” y sus diferentes significados como algo construido para hacer frente a la discriminación y poder luchar contra la huella del estigma social del sinhogarismo y la pobreza. Un planteamiento como el desarrollado en esta investigación, basado en las biografías y voces de las propias personas que viven estas experiencias, permite alejarnos de las categorizaciones negativas de carácter simbólico-conceptual que comportan procesos estigmatizadores y prejuicios basados en la alteridad. Este abordaje puede ser incluido en la planificación de otros programas *Housing First* con el objetivo de que no dependa únicamente de la visión que la persona tenga de sí misma, y que la transformación respecto a la estigmatización sea entendida en un sentido amplio, que involucre las personas, en las instituciones y en la sociedad.
