# Supplementary material for: Estigma en personas sin hogar y atención en salud: reflexiones desde un programa Housing First en Barcelona, España
Source: Salud Colect. 2024 Jun 5;20:e4826. [Article in Spanish] doi: 10.18294/sc.2024.4826 (PMC11925747; doi:10.18294/sc.2024.4826)
Supplement: Translation

# Translation: Stigma in homelessness and health care: reflections from a Housing First program in Barcelona, Spain

**Authors:** Marta Llobet-Estany, Mercedes Serrano-Miguel, Araceli Muñoz, Adela Boixadós-Porquet, Belén Campomar

**Affiliations:** 1 Doctora en Sociología. Docente e investigadora, Unitat de Formació i Recerca - Escola de Treball Social, Universitat de Barcelona, España. mllobet@ub.edu; 2 Doctora en Antropología Social. Docente e investigadora, Unitat de Formació i Recerca - Escola de Treball Social, Universitat de Barcelona, España. mercedes.serrano@ub.edu; 3 Doctora en Antropología Social. Docente e investigadora, Unitat de Formació i Recerca -Escola de Treball Social, Universitat de Barcelona, España. aracelimunoz67@ub.edu; 4 Licenciada en Sociología, diplomada en Trabajo Social. Docente e investigadora, Unitat de Formació i Recerca - Escola de Treball Social, Universitat de Barcelona, España. aboixados@ub.edu; 5 Licenciada en Relaciones Internacionales, Especialista en Política, Evaluación y Gerencia Social. Investigadora, Universitat de Barcelona, España. belen.campomar@gmail.com

**Keywords:** Homelessness, Social Stigma, Healthcare System, Spain

## Abstract

The experience of homelessness is associated with strong stigmatization processes, which are often reflected in the treatment received from professionals and the healthcare system itself. This article aims to analyze the experiences of participants in a program for homeless individuals in Barcelona called Primer la Llar within the healthcare system, and how the stigma they suffer affects the care processes. This program follows the Housing First model, a social intervention that proposes providing housing without preconditions to individuals with long histories of street living, who suffer from severe mental disorders and/or addictions. Based on individual interviews with 20 participants conducted between 2016 and 2020, it is observed that in certain cases, entering the program, having housing availability, support from professionals, and the development of their own strategies had positive effects on improving their health, although they continue to perceive discriminatory attitudes in some medical settings. It is suggested that the transformation regarding stigmatization be understood broadly, affecting individuals, institutions, and society as a whole.

## INTRODUCTION

The problem of homelessness has experienced a sustained growth in statistical terms over the past few years in different parts of the world. In the case of the European Union, it is estimated that in 2023 there were at least 895,000 people sleeping on the streets, staying in night shelters, or in temporary accommodations, showing an increase in most member countries compared to previous years^1^. In Spain, the National Institute of Statistics reported that, in 2022, more than 28,000 homeless people were being attended in assistance centers, which represents an increase of 24% compared to 2012^2^. This number only represents the people attending these facilities, so the total figure is estimated to be even higher. For Barcelona, the context in which this research is conducted, data from 2022 indicates the existence of 4,197 people sleeping on the streets, in settlements, or using municipal resources, which is 98% more than the first report in 2008^3^.

In all cases, these are people who face daily violations of their fundamental rights, being totally or partially excluded from the possibility of having their basic needs met: housing, food, education, and good health, among other essential needs for a dignified life. Thus, access to basic goods and services is obstructed by their condition of extreme poverty-lack of economic resources to acquire them-but also by the social construction generated around their existence, being socially marked by stigma, discrimination, and isolation^4^^,^^5^^,^^6^. At the same time, the experience of homelessness is associated with processes of strong stigmatization that, on many occasions, are reflected in the treatment people receive from professional teams and the healthcare system itself. Thus, although Spain has universal, public, and free healthcare services, other non-economic barriers are deployed that hinder access to healthcare facilities, even creating distance and rejection^7^^,^^8^^,^^9^^,^^10^. This situation reinforces precarious conditions, health difficulties, and increased risk of death^11^^,^^12^^,^^13^^,^^14^.

Housing First is a social intervention with a long international trajectory but an emerging development in the Spanish context. It proposes providing housing without preconditions to people with long histories of living on the streets, suffering from severe mental disorders and/or addictions. This article aims to analyze the experiences of participants in the *Primer la Llar* program as users of the healthcare system and with the professionals who attended to them. The objective is to identify the effects that entry into the program, the availability of housing, and the support of the *Primer la Llar* technicians had on the process of care and health improvement. Additionally, it explores the influence that this circumstance had on the attitudes of the healthcare professionals who attended them. Furthermore, the study examines the legal strategies implemented by the individuals themselves in the pursuit of their care and health improvement. Overall, this study provides results from an experience in southern Europe, a context different from where Housing First programs are usually evaluated, thereby enriching the availability of evidence for the model in new territories.

### Housing First Model and its Application in Barcelona: *Primer la Llar* Program

The Housing First model is a program that has generated innovative practices for assisting people with long histories of homelessness, severe mental disorders, and/or addictions, for whom the usual assistance circuit had not provided an adequate response^15^. It begins by providing permanent and independent housing, considering it a basic right and a facilitator of recovery. Unlike the traditional Staircase or Treatment First model, entry into housing is not conditioned on a specific treatment, and the person decides what steps to take to enhance their well-being and community integration^16^^,^^17^.. Participants have a support team, and there are two types of approaches regarding this aspect. On one hand, there is the Assertive Community Treatment (ACT) for cases of people with severe mental disorders, with a multidisciplinary team that meets daily to assess risks and needs, covering 24 hours a day, seven days a week^18^^,^^19^. On the other hand, there is the Intensive Case Management (ICM) model for people with less frequent treatment needs, involving weekly home visits and the use of community services for psychiatric or medical treatments^20^. Since the pioneering experience of Pathways to Housing, led by Sam Tsemberis in New York in the 1990s, the Housing First model has spread^21^^,^^22^ and has been evaluated from multiple theoretical and methodological frameworks, mainly in the USA, Canada, Australia, and also in some European countries^17^^,^^18^^,^^19^^,^^23^^,^^24^^,^^25^^,^^26^^,^^27^^,^^28^^,^^29^.

In terms of housing, Spain is characterized by significant access difficulties for a substantial portion of its population. Although it has a large residential stock, this does not meet the needs of low-income groups, especially those who cannot access rental housing in the real estate market and who require social housing^30^. With a social housing index of 2.5% of main residences, Spain ranks as one of the countries with the lowest social housing rates within the European Union (EU), far below the European average of 9.3%^31^. Furthermore, compared to other countries in the region, Spain is the EU territory where renters allocate the highest percentage of their income to cover housing costs^32^. In this context, in 2015, the Barcelona City Council became the first Spanish public administration to implement the Housing First model with the pilot program *Primer la Llar*. The program provided supported housing to 50 people experiencing homelessness^33^. It selected individuals with long histories of street life, mental disorders, and/or addictions, functional autonomy for daily living, and the availability of income or the capacity to obtain it. From this experience, follow-up and evaluation were conducted using a qualitative and longitudinal approach, from which this article emerges.

### Health Conditions in Homelessness Trajectories and the Role of Stigma in Access to the Healthcare System

Social factors are a determinant of health, which also influences the possible ways of seeking social support in both formal and informal settings^34^^,^^35^. In this regard, this study adopts an expanded concept of health^36^^,^^37^ that includes the biological dimension, but also the social, cultural, psychological, and political factors that influence people’s health status, thus avoiding a reductionism more typical of the biomedical model. This concept of health is neither univocal nor universal, “like all linguistic practices, health is metaphorical: it absorbs and expresses a variety of meanings found throughout culture”^38^. This suggests the relevance of conducting qualitative studies, with approaches centered on people and their contexts to propose a respectful analysis that assumes a broad understanding of the lived sufferings and the consequent care itineraries that people have gone through. Likewise, working with narratives has led us to adopt the analysis of health problems based on the concept of malaise^39^, through which we have been able to approach the lay strategies used by participants to address health issues.

People with long histories of homelessness often have poor physical and emotional health. Morbidity and mortality rates are generally higher than for the rest of the population, especially among young people and women^8^^,^^10^^,^^40^^,^^41^. Additionally, not having a home increases symptoms of mental health problems and drug use^42^^,^^43^, and paradoxically, despite needing more healthcare, they often have less access to care^44^^,^^45^. In Spain, various studies corroborate this fact, which occurs in different cities and care centers^14^^,^^46^^,^^47^^,^^48^. Housing First approaches often yield positive results on the health conditions of people who access these programs. In this regard, various studies show the crucial role of adequate housing for people’s emotional well-being^49^^,^^53^, as well as how having a living space of their own can improve the effectiveness of treatments by allowing them to follow routines without the stress caused by homelessness^16^. It is argued that having housing facilitates the recovery process since the home is where a person can be themselves, apart from the outside world, where identity is created, routines are established, and daily life develops^54^^,^^55^^,^^56^. However, from this perspective, recovery is not a fixed goal common to all people but rather a horizon adapted to subjectivity and individual characteristics. Following Leamy et al.^57^, recovery is a journey, configured by dimensions and composed of different phases. It is a complex concept that is useful as long as it does not impose specific characteristics or unrealistic expectations. Palimaru et al.^58^ point out that the Housing First model, compared to other homelessness approaches, is more effective in terms of the provision, coordination, and use of health services, but nevertheless,

…...Housing First and non-Housing First programs face a series of systemic challenges, including the complex and slow nature of working with high-needs clients, inadequate mental health services, case manager turnover, and staff shortages, which can undermine some of the benefits of the Housing First approach^58^.

Stigma, understood as an individual condition that hinders full social acceptance^59^, is an essential element to comprehend recovery processes, which are particularly complex for individuals facing extreme vulnerability due to factors such as poverty, mental health issues^60^^,^^61^, addictions^62^, and/or disabilities, among others, leading to multiple forms of stigma ^(^^63^.

The literature indicates a tendency to place these individuals within a passive citizenship framework, perceiving them as dependent on the state both economically and socially, thus stripping them of their agency. In contrast, those who are independent are often seen as active and full citizens^64^. The roots of these discriminatory behaviors lie in the structural parameters of societies that define norms and exclude those who deviate from them. Power dynamics among different actors within a community become crucial in enforcing these behaviors^60^.

Link and Phelan^65^ point out the existence of a power of stigma, given that:

...stigmatizers have strong motivations to keep people repressed, inside or outside, and the best way to achieve these goals is through stigmatization processes that are indirect, widely effective, and hidden in culturally assumed circumstances^65^.

Stigmatizing mechanisms operate widely among individuals experiencing homelessness^4^^,^^5^^,^^6^, leading to various consequences such as increased isolation, difficulty in forming social bonds, and navigating institutions^9^^,^^65^. For instance, Belcher and DeForge^4^ highlight the community rejection that arises when the construction of a homeless shelter is announced, as residents in the neighborhood do not want this population nearby, identifying them as problematic and undesirable. Consequently, many homeless individuals acknowledge feeling stigmatized both by society at large and within healthcare facilities^66^^,^^67^^,^^69^.

Multiple studies address the connections between individuals experiencing homelessness, mental health issues, and/or addictions, and their experiences within the healthcare system, identifying stigmatizing situations and the resulting consequences for this population’s trajectories^8^^,^^10^^,^^13^^,^^69^. Some of the attitudes encountered from medical professionals include: infantilization; lack of respect and interest^60^; the belief that they present to emergencies seeking narcotics prescriptions, based on the stereotype that they are addicts^70^; and blaming comments regarding their lack of health care^71^, among others. Additionally, they also face a preliminary barrier from administrative or security staff, who may also harbor prejudices and discriminatory actions^11^. In some cases, additional factors exacerbate this situation, such as homeless migrants facing even greater difficulties and inequality in accessing healthcare^14^. These discriminatory experiences not only result in inadequate access to treatments but also directly impact health through the stress they induce in those exposed to such situations, potentially causing physical and mental illnesses^12^.

The flip side of the same reality is the significantly supportive role that healthcare team relationships can play in recovery processes^58^^,^^72^^,^^73^^,^^74^. Padgett et al.^9^ analyze the conditions that facilitate or hinder treatments for homeless individuals with severe mental health issues or substance use, identifying that one facilitating aspect is “acts of kindness” from medical staff, where they display humanity and warmth, breaking away from the norm of routine and dehumanizing encounters they often face. In this regard, Fang et al.^5^ employ the concept of “cultural humility,” suggesting that healthcare professionals, through self-reflection, become aware of their influence in these relationships, enabling them to empathize with the beliefs and experiences of those they serve and adapt to their particularities. They understand that a singular cultural perspective cannot be universally applied to all individuals.

## METHODOLOGY

### Design, Population, and Sample

This research employed a qualitative methodology^75^, from an interpretative perspective, using a longitudinal design. Adopting a narrative approach allowed for the emergence of complex elements to understand the experiences of the program participants, while the longitudinal perspective enabled the observation of the program’s impact on individuals’ lives over time, following international research practices^26^^,^^76^^,^^77^^,^^78^. The study was structured into three phases spanning from 2016 to 2020. In each phase, semi-structured interviews were conducted with the participants^79^, designed and carried out by a research team composed of professors from the Unitat de Formació i Recerca - Escola de Treball Social, Universitat de Barcelona, who are responsible for the authorship of this article.

The *“Primer la Llar”* program included a population of 50 individuals, out of which 20 were part of the continuous and final sample of this research (n=20), representing 40% of the population. Compared to other studies with similar characteristics, this is considered a sizable sample^80^. The sample selection was intentional, maintaining 90% of the original sample size, with an additional reserve of three people in case of dropouts during the research period. The average age of the participants was 55 years. Table 1 outlines the characteristics of the final sample, including gender, origin, initial diagnosis, chronicity (defined by the U.S. Department of Housing and Urban Development^81^), and marital status.

**Table 1:** Sample Characteristics (n=20). Barcelona, 2016 and 2020.

Variables	n	%
Gender		
Men	16	76
Women	4	69
Origin		
Native	14	70
Community migrants	1	5
Extracommunity migrants	5	25
Initial Diagnosis		
Addiction	6	30
Dual Pathology	3	15
Mental Disorder	7	35
Other/Unknown	4	20
Chronicity*		
1 to 5 years	8	40
6 to 10 years	7	35
More than 10 years	5	25
Marital status		
Separated	10	50
Single	6	30
Divorced	3	15
Married	1	5

Source: Own elaboration. * Chronically homeless defined by the U.S. Department of Housing and Urban Development, 2015^81^.

### Data Collection and Analysis

The fieldwork was conducted in three phases, with interviews conducted at the beginning, 24 months, and 40 months into the program, designing and adapting scripts for each phase of the research. The first phase, conducted from May 2016 to June 2017, focused on understanding the participants’ stories upon entering the program and their life trajectories during that previous period. This involved exploring the life processes they had undergone, including experiences that had caused them significant suffering, their housing trajectories, and their prior contacts with specialized social services. The second phase, conducted from May 2018 to January 2019, began after participants had been in the program for two years. Its objective was to understand their adaptation process to new housing and the course of their personal and social recovery experienced since entering the program. In the third and final phase, conducted between September 2019 and January 2020, the focus was on understanding the changes experienced after three years in the program, as well as the self-evaluation that the participants had made regarding these changes and their impact on their current state. The duration of the interviews ranged from 2 to 5 hours, depending on whether they were conducted in one or two sessions. They were primarily conducted in public spaces (cafés, municipal centers, open spaces), and in some cases, at participants’ homes, based on their availability and expressed comfort.

The fieldwork was tailored to the needs of the interviewees. Initially, contact was established through the program’s professional team, allowing for a mutual assessment of the best timing for conducting the interviews. In subsequent stages, direct contact was made with the interviewees. Recordings were transcribed verbatim, and codes were developed based on the study’s questions and objectives, which were adjusted to the themes incorporated in each phase. Additionally, emergent codes agreed upon by the research team were added, and peer review was conducted at each stage. The analysis was conducted using Atlas.ti 8.

### Ethical Considerations

This research adhered to the Ethical Code of Integrity and Good Research Practices of the Universitat de Barcelona, established in 2019. Each participant voluntarily agreed to take part in the research, with a commitment to use the data solely for research purposes in accordance with Organic Law 15/1999 and the Law on Protection of Personal Data and Guarantee of Digital Rights - Organic Law 3/2018 of December 5. Every interviewee was verbally and in writing informed about the research objectives, provided with and signed an informed consent form, and received a copy of the interview transcripts at each phase. Anonymity was ensured by using pseudonyms in this article, and strict confidentiality of data was maintained.

## RESULTS

### Main Effects of the *Primer la Llar* Program on Health Trajectories

The first phase of the research revealed that participants suffered from significant physical health problems, identifying diseases with varying levels of severity. Among the milder conditions mentioned were dental problems, hearing and vision impairments, diabetes, cholesterol issues, and blood pressure fluctuations. More severe conditions included cancer, back injuries, and hepatitis C. According to the narratives, these illnesses were exacerbated by their life journeys, precarious living conditions, alcohol consumption, lack of access to healthcare resources, and in some cases, aging itself, such as with lumbar pain or neurodegenerative diseases. In the second and third phases of the research, we were able to observe that, for the vast majority of interviewees, the change in housing situation had led to significant improvements in their health status.

*“Of course it helps me. It’s not the same being on the street as being under a roof. If I were on the street, I don’t even know how I would be”.* (Miguel Ángel, interview phase 2)

*“Better than before. Before, I used to drink a lot, but not anymore. When I was on the street, I drank a lot. Even though the doctor prescribed Antabuse, I would stop taking it... well, everything was very bad. The diabetes was the same. With diabetes, I drank, smoked... a disaster. Now it’s better, since I have the apartment, everything is more controlled”*. (Omar, interview phase 2)

*“Man, the apartment has helped me a lot. If I want to stretch out, I stretch out. If something hurts, I can sit down. It helps me a lot. It’s the foundation of good health. If you’re in a shelter and want to take a nap, they won’t let you. You have a headache, they won’t let you; you have a fever, they won’t let you. Many things... I’ve seen people who have had a hard time. But here at home, you can live your life [...] medicine, you take care of your health. The home is the foundation”*. (Youssef, interview phase 3)

One issue that emerged from the narratives was the oral health problems and the physical and mental repercussions they had caused individuals in the context of poor and inadequate nutrition, leading to infections and digestive complications, among other physical issues. At the same time, in the most extreme cases, it was perceived as a health problem that exacerbates and makes poverty even more visible, operating on a symbolic level that reinforces the stereotype of homelessness and contributes to social isolation, low self-esteem, and communication difficulties.

*“Because deep down I thought, since I’m here, since I’ve taken the step, at least to have a social life, when I’m completely well, when I have my teeth, when... Because I’m still a tooth junkie, you know?”* (Alfonso, interview phase 3)

Regarding experiences of mental distress, during the interviews there was hesitation to discuss them, especially when approached using medical terminology. However, in the narratives, there was an implicit acknowledgment of the potential mental suffering that could arise as a result of life on the streets.

*“Since I’ve been out on the street... that’s when my mind started to work differently. When things didn’t go the way I wanted, I would shut down, I would block myself. I couldn’t find another way out.”* (Maria José, phase 2 interview)

In some cases, such as in the interview with María José, there is a significant underlying background of pain and suffering, regardless of whether there is a clinical diagnosis or not. Thus, in the narratives, years of suffering, anguish, having “gone through a lot”, feeling “a very deep discomfort inside” and “not wanting to revisit” are mentioned, acknowledging the experience of a personal process of suffering where there is a preference to “live in the present”, avoiding confronting the possible distress experienced and its causes. On the other hand, it is recognized that access to housing has provided them with a situation of greater tranquility, security, stability, and increased self-esteem, which has positively influenced their physical and psychological well-being.

*“Well, to value things more, to feel good about myself... to be able to do whatever I want, right? It’s what I told you before. You don’t depend on anyone, nobody is telling you that you have to do this or that... and you can be autonomous*”. (Miguel Ángel, phase 2 interview)”

On the other hand, in the third phase of the research, it is observed that housing and the temporal limitations imposed by the program present a landscape of uncertainties that individuals must contend with. Thus, anxiety and distress emerge regarding the potential future housing situation. This situation ultimately becomes one of the major sources of discomfort reported by the interviewees, with particular consequences for mental health.

*“I live peacefully, but I don’t know what will happen tomorrow.”* (María, phase 3 interview)

### Access to the Healthcare System: Change or Continuity?

In the first phase of the research, accounts were gathered regarding the functioning of the healthcare system for the homeless population - that is, their experiences prior to entering the program - which allowed for the identification of various issues: lack of coordination between hospitals and social services network facilities, administrative requirements, and long waiting times to access services. The most critical evaluations regarding access or aspects of the functioning of some health facilities were primarily made by drug users. For instance, there were questions about the requirement to be registered at a fixed address in order to access methadone treatment. This administrative criterion, which may be logical for organizing services according to the user population in each area of the city, can act as a deterrent barrier to treatment access for those unable to meet this requirement.

Additionally, concerns were raised about the operating hours of certain services, such as venipuncture rooms, which are part of harm reduction policies and programs. As highlighted in the narratives, drug users may not necessarily adhere to the same schedules and guidelines in their consumption patterns.

In the second and third phases of the research, it was found that, once participants had housing through the *Primer la Llar* program, they continued to experience difficulties in accessing healthcare due to institutional practices such as staff changes or rotations. These practices hinder the establishment of stable relationships and may lead to mistrust among the participants.

*“Yes, when it comes to [seeing the] psychiatrist, they have changed my psychiatrist three times since I’ve been there... So I tell them the same story every time...”* (Miguel Ángel, phase 2 interview)

It was also evident how the stigma associated with mental disorders can act as a barrier to attending a mental health professional’s appointment, as the utility of treatment may not be fully understood and could be perceived as a proposal that might lead to rejection.

*“I used to go to a psychologist, but I stopped when I came here. You want to get me into the worst... I used to go to one whose goal was to find out if I was a whore, if I smoked, etc., then to another... They won’t trick me again with these people. You won’t trick me again with these people. I don’t want to know anything about them. I won’t go, you won’t trick me again with these people. I won’t go, I won’t go back”.* (María, phase 2 interview)

*“Yes, but right now I’m not ready to go again either. Because I know that when I go, they will make me remember, they ask me questions, and now... I say, let’s leave it like that. Maybe later on I will go because everything will hit me at once and too much happiness isn’t good either. I know that. All this good... then has its ups and downs”*. (María José, phase 3 interview)

However, positive experiences also emerge that demonstrate the practical utility of a psychotherapeutic space, and how building trusted relationships between parties can support the recovery process of those receiving treatment.

*“Yes, I’m seeing a psychiatrist and it’s going quite well because I get a lot off my chest. I mean, I say things that I might not dare to say on the street.”* (David, phase 3 interview)

In different accounts, it is evident how housing facilitated attendance at medical appointments and the follow-up of proposed treatments. In fact, all interviewees mentioned the Primary Care Center (CAP) as their main healthcare facility and the hospital as the service used in case of emergencies.

*“I have suffered on the street for many years and to have an apartment, your key, your house, to do whatever you want, go out whenever you want, come in, it’s like winning the lottery. For me, it’s a great joy. If you’re on the street, you get worse and you don’t have time to follow the medicines, the medication, you don’t feel like doing anything... you have to be like an animal lying around. It’s not like at home, where you clean, find motivation, and it gives you strength to do things.”* (Said, phase 2 interview)

Regular contact with the medical team at the Primary Care Center (CAP) and with the neighborhood pharmacy has allowed them greater access to medications as a therapeutic measure, which is highly valued by the majority of interviewees as a way to alleviate physical and mental pain. Additionally, most participants have perceived a direct benefit by experiencing significant improvement in the management of certain symptoms. Indirectly, this approach seems to be seen as a method to potentially avoid psychotherapeutic intervention and the consequent addressing of episodes of intense psychological pain.

In some narratives, there was also an observed evolution in the relationship between participants in the *Primer la Llar* program and healthcare personnel, highlighting an improvement in the quality of trusted relationships and support, which positively impacts the health status of individuals.

*“They have all been very clear: they have spoken with me, they have explained things to me without shouting, which is how I like things. They have explained things to me very well, both the pulmonologist and Mr. Tomás, the GP. They have treated me like everyone else, like a person, not like cattle”*. (Fermín, phase 2 interview)

Indeed, interviews indicate that there was closer and more continuous contact with some members of the professional team; for instance, nursing staff are appreciated for being more accessible in practice and are often the ones with whom participants maintain more regular visits. Similarly, the person attending at the neighborhood pharmacy, who can be accessed relatively frequently and in a less formal manner, is also mentioned. Despite often lacking specific training in the social field, these individuals can become part of a care structure that serves as a reference point.

However, even though having housing brings them closer to leading a socially accepted life and gives them more capacity to claim their rights, in some cases they perceive that they still carry the stigma of having been homeless. In this way, they find it very challenging to integrate into the society they now live in after having been outside of it.

*“To them, I continue to be just another homeless person, so to speak; with them, I am a user on standby. I have always seen it this way.”* (Alfonso, phase 3 interview)

They also perceive that they are still marginalized, and often receive differential treatment compared to the rest of the population, feeling segregation and exclusion from certain spaces where existing prejudices and stigmatizing discourses continue to label them, justifying discriminatory practices and actions towards them.

“Miguel Ángel: *Without looking at your past, just because of the past you’ve had, they label you as if you were a weirdo*.

Interviewer: *Have you felt that sometimes and do you think it would be better if...*

Miguel Ángel: *I felt that there at [name of mental health association*].” (Miguel Ángel, phase 3 interview)

#### Beyond institutions: Self-care strategies for health

The narratives also revealed that participants in the program had developed strategies related to certain types of alternative therapies, self-managing their treatment, and asserting themselves with healthcare professionals. These were skills learned and developed from their own life experiences. For example, they turned to meditation or practiced Buddhism as means of relaxation and spirituality, called their families for reassurance, or even accepted delusions as part of their personal functioning. Some strategies were related to community and neighborhood facilities, such as joining a gym to compel themselves to leave home and confront social phobia. Others involved using their home differently, such as getting more sleep, or using natural remedies that were not accessible while living on the street.

*“I don’t know, I joined the gym, because, obviously, I wasn’t leaving the house. I felt safe locked up here, because I developed a phobia. When I say phobia... I didn’t feel like seeing people. And now it’s also hard for me. And taking the subway, but now, since I have to take it every day to go to the Clinic... So I joined the gym and it’s going well for me”*. (Silvia, phase 2 interview)

Experiences of suffering have led to increased self-awareness regarding the effects of treatments and greater confidence and agency in relation to medical proposals. Thus, in the final phase of the research, self-management practices of medication have also been observed.

*“Well, I don’t know, it depends on how I feel too. There are days when, if I go without it for a few days, my body itself craves it, it craves those substances, that includes morphine and of course the body needs that, it’s a substance the body needs. It’s like the morphine patches they give to smokers. Not smokers, no, morphine patches for people in pain. That’s why I need it, but I don’t need it daily. When I feel bad, when I feel yawning, I take it, for example today I had to take it, I took it this morning, but maybe tomorrow I won’t take it.”* (Miguel Ángel, phase 3 interview)

Regarding social support, in some cases, it has been evidenced how the primary contact person at *Primer la Llar* becomes the accompanying or supportive figure during health system visits, chosen by the participants as someone they trust. This is done with the intention of ensuring that healthcare professionals listen more attentively to their situation.

*“I really like going with the reference 32, because she always reminds me of something. And if not, she says it, because she has free rein to say whatever she wants. Everyone who accompanies me to the doctors, everyone, has free rein to say whatever they want, in case I forget something.”* (Fermín, phase 2 interview)

Similarly, housing has become, in some cases, a space for care and/or self-protection where individuals can seek refuge when feeling unwell. This has allowed them to have greater control over activities related to self-care and household tasks. In narratives, the importance of being self-sufficient in performing these tasks is highlighted, and how their self-care has not only been beneficial for their physical health but also for their mood, indicating that it has boosted their self-esteem. Moreover, many participants have found that having housing has helped improve their eating habits and well-being: they have improved their weight and physical condition, seen improvements in certain diseases (cholesterol, diabetes), and have the ability to decide what they eat at appropriate times.

*“Maybe on the street, I would eat once a day, and here I eat four or five times. Everything has changed.”* (María José, phase 2 interview)

Another health-related habit that has been regained through having housing is hygiene and the desire to improve their personal appearance. The means through which they aim to achieve this improvement include physical exercise, clothing choices, and dental care treatments.

*“When you arrive at a place like this apartment, just seeing the house and being here makes you want to be able to take a shower.”* (Manuel, phase 3 interview)

These strategies have also been used for coping with addictions among participants who have reported using substances (mainly alcohol and marijuana, but also tobacco). The desire to reduce or eliminate this consumption has led to the gradual adoption of coping mechanisms, some based on the guidelines provided by the professional team, such as attending the Center for Care and Monitoring (CAS) for substance dependence or taking specific medications (like Antabuse for alcohol). In other cases, coping has been achieved through habit changes, such as altering friendships, relationships, or modifying certain consumptions in favor of less harmful ones.

In this regard, having housing also allows for a certain level of limitation and control, and the establishment of healthier lifestyle habits. However, some individuals mention difficulty in abandoning street life routines, such as poor eating habits, and due to economic reasons, they continue to rely on soup kitchens or meal vouchers instead of eating at home in a healthier manner.

## DISCUSSION

The narratives of the participants in the *Primer la Llar* program have provided insight into their lived experiences regarding health status and access to care after obtaining housing. They have also allowed us to understand their perceptions of their interactions with healthcare professionals and the development of personal care strategies outside of institutional settings.

Firstly, this analysis confirms that recovery processes are complex and diverse^57^, just like the participants and their trajectories. It is observed that their health statuses and approaches to engaging with professionals, treatments, therapies, and the healthcare system are equally varied. Far from seeking to identify a uniform pattern or a singular answer, qualitative and interpretative methodology allows us to delve deeper into the multiple lived experiences.

Regarding their health status, a series of ailments and pre-existing conditions prior to entering the program have been identified, similar to those indicated by other studies and prevalent among individuals experiencing homelessness^14^^,^^40^^,^^41^^,^^42^^,^^43^^,^^46^^,^^47^^,^^48^. Participants explain that while homeless, managing their symptoms was either scarce or subject to very sporadic monitoring. Based on their experiences, the lack of a stable housing situation, potential fragility of their social networks, and difficulties in accessing information have been crucial factors contributing to this disadvantaged situation. Over the course of the research and subsequent processes observed, it is noted that in some cases, housing progressively becomes a stabilizing force, helping to mitigate the detrimental effects that street life or previous assistance programs may have had on people’s health. Throughout these four years, the relationship between program participants and the professional team in healthcare services, or personnel within this context such as pharmacy attendants, has strengthened. In many cases, the contact has become closer over time, considering the increase in health tests and regular monitoring conducted.

However, the difficulties that pose obstacles to the recovery of participants in the program are often consequences of their entire life journey. This journey has been characterized by economic exclusion and health problems, but also by a daily life different from that of the majority of people, and an often complicated relationship with institutions tasked with responding to those experiencing social exclusion. At this point, the variable of stigma comes into play, specifically analyzing the effects of this discriminatory attitude generated by healthcare teams towards individuals who are or have been homeless, and who may also have mental health and/or addiction issues. This action further impedes access to medical visits and constitutes a painful experience in itself. This relationship, documented in other studies^8^^,^^10^^,^^11^^,^^12^^,^^13^^,^^69^^,^^70^^,^^71^, was identified in some participants in this research, sometimes explicitly and at other times more indirectly. For instance, participants mention going to appointments accompanied by a program representative because they believe they will receive better care that way. Participants’ narratives have highlighted these experiences also in relation to other institutions or social spaces. It is evident that the stigma of homelessness remains a barrier hindering full citizenship and social integration.

Alongside identifying material and symbolic barriers within the healthcare system, the participants in the program have been observed developing their own strategies to self-manage various aspects of care. Some of these strategies were already practiced when they were homeless, while others are new practices made possible by having housing, generally linked to achieving greater autonomy. These strategies are evident in narratives discussing ways to navigate difficult days, distressing situations, or physical pain. They involve managing medications as well as practices associated with relaxation, spirituality, and care in nutrition and hygiene. While the ability to use personal tools for managing discomfort is highly beneficial, the framework of this analysis does not intend to interpret it solely as the creative development of strategies to adapt to severe rights violations. Such a perspective risks focusing on individual responsibility, encapsulating the issue within personal aspects, and diminishing the actual weight of homelessness and stigma at social, collective, and political levels^60^. It is essential that each person’s agency extends beyond individual strategies to encompass group and collective efforts, ensuring these strategies are not isolated and fragile. Most interventions and activities during the *Primer la Llar* program were individually focused, with few opportunities for group work on these aspects. Achieving recovery requires a supportive context that facilitates growth, and interventions should aim to influence this context to create better conditions of existence^4^. Consistent with the literature presented earlier, this necessitates collective awareness efforts and recognition of dual processes: one individual and the other communal, identifying cultural barriers and the responsiveness of services^5^.

Regarding the limitations of the research framing this article, several points are noted. Firstly, conducting fieldwork with individuals who have extensive histories of homelessness and multiple experiences of rights violations presents challenges. At times, this engenders a degree of mistrust towards the research process, the researchers themselves, the interviews, and the act of recounting their life stories once again, often painful ones. This can manifest in declines in participation, with participants expressing fatigue towards interviews, feeling unheard, and experiencing repetition and over-observation. This limitation acknowledges the increased likelihood of sample fatigue common in longitudinal research. Secondly, it is noted that the sample selection was influenced by the timing of individuals’ entry into the program, resulting in a higher proportion of participants who joined at a later stage. Finally, the innovative nature of the program places us in an experience where there are many theoretical references but few practical ones.

## CONCLUSIONS

This study highlights that individuals with long histories of homelessness, experiencing severe mental disorders and/or addictions, can find in a program like *Primer la Llar* new opportunities for healthcare and engagement with the healthcare system. Having housing has enabled them to adopt basic daily care practices and habits that have become healthy routines, and provided them with a space where they can feel emotionally well. However, some narratives have identified that access to healthcare services remains limited due to discriminatory attitudes, burdened by the social stigma associated with having lived on the streets.

In light of this reality, it is evident that beyond implementing strategies to address homelessness focused on individuals experiencing this situation, there is a need to rethink how institutions, organizations, and the community at large engage with this population. It is crucial to continue reflecting on the concept of “normalcy” and its various meanings as a constructed framework to combat discrimination and confront the social stigma associated with homelessness and poverty.

An approach like the one developed in this research, based on the life stories and voices of those experiencing these situations, allows us to move away from negative symbolic-conceptual categorizations that perpetuate stigmatizing processes and prejudices based on otherness. This approach can be integrated into the planning of other Housing First programs with the goal of not solely relying on individuals’ self-perception, and ensuring that the transformation regarding stigma is understood in a broad sense involving individuals, institutions, and society as a whole.
